# Deregulated Expression of Immune Checkpoints on Circulating CD4 T Cells May Complicate Clinical Outcome and Response to Treatment with Checkpoint Inhibitors in Multiple Myeloma Patients

**DOI:** 10.3390/ijms22179298

**Published:** 2021-08-27

**Authors:** Anna Kulikowska de Nałęcz, Lidia Ciszak, Lidia Usnarska-Zubkiewicz, Irena Frydecka, Edyta Pawlak, Magdalena Szmyrka, Agata Kosmaczewska

**Affiliations:** 1 Hematology and Hematological Oncology Department, Provincial Hospital, 45-372 Opole, Poland; a_masternak@poczta.fm; 2 Hirszfeld Institute of Immunology and Experimental Therapy, Polish Academy of Sciences, 53-114 Wroclaw, Poland; lidia.ciszak@hirszfeld.pl (L.C.); irena.frydecka@umed.wroc.pl (I.F.); edyta.pawlak@hirszfeld.pl (E.P.); 3 Department and Clinic of Haematology, Blood Neoplasms, and Bone Marrow Transplantation, Wroclaw Medical University, 50-367 Wroclaw, Poland; lidia.zubkiewicz@gmail.com; 4 Department of Rheumatology and Internal Diseases, Wroclaw Medical University, 50-367 Wroclaw, Poland; magdalena.szmyrka@umed.wroc.pl

**Keywords:** multiple myeloma, CD4 T cells, PD-1, CTLA-4, checkpoint inhibitors, clinical outcome

## Abstract

Unlike solid-tumor patients, a disappointingly small subset of multiple myeloma (MM) patients treated with checkpoint inhibitors derive clinical benefits, suggesting differential participation of inhibitory receptors involved in the development of T-cell-mediated immunosuppression. In fact, T cells in MM patients have recently been shown to display features of immunosenescence and exhaustion involved in immune response inhibition. Therefore, we aimed to identify the dominant inhibitory pathway in MM patients to achieve its effective control by therapeutic interventions. By flow cytometry, we examined peripheral blood (PB) CD4 T cell characteristics assigned to senescence or exhaustion, considering PD-1, CTLA-4, and BTLA checkpoint expression, as well as secretory effector function, i.e., capacity for IFN-γ and IL-17 secretion. Analyses were performed in a total of 40 active myeloma patients (newly diagnosed and treated) and 20 healthy controls. At the single-cell level, we found a loss of studied checkpoints’ expression on MM CD4 T cells (both effector (Teff) and regulatory (Treg) cells) primarily at diagnosis; the checkpoint deficit in MM relapse was not significant. Nonetheless, PD-1 was the only checkpoint distributed on an increased proportion of T cells in all MM patients irrespective of disease phase, and its expression on CD4 Teff cells correlated with adverse clinical courses. Among patients, the relative defect in secretory effector function of CD4 T cells was more pronounced at myeloma relapse (as seen in declined Th1/Treg and Th17/Treg cell rates). Although the contribution of PD-1 to MM clinical outcomes is suggestive, our study clearly indicated that the inappropriate expression of immune checkpoints (associated with dysfunctionality of CD4 T cells and disease clinical phase) might be responsible for the sub-optimal clinical response to therapeutic checkpoint inhibitors in MM.

## 1. Introduction

Multiple myeloma (MM) is an incurable hematologic malignancy characterized by proliferation and accumulation of clonal plasma cells producing M-protein in the bone marrow (BM) [1]. A biologic property of MM is progressive development of immune deficiency that leads to tumor escape, disease growth, and clinical complications, such as bone disease or recurrent serious infections [2]. The pathogenesis of the immune dysregulation in MM is complex and includes disease- and treatment-related factors, thus resulting in cumulative immunosuppression and increased risk of severe infections. The immune dysfunction in MM is associated with the inhibition of normal plasma cells with subsequent hypogammaglobulinemia as well as impaired cellular immunity, including dysfunction of T cells, dendritic cells (DCs), and NK cells [2,3]. The profound T cell alterations in MM include a rapid loss of effector function and an increase in the abundance of immunosuppressive Tregs in the BM [2]. A major role in the development of the immunosuppressive state in MM has recently been attributed to the immune checkpoints, such as PD-1, CTLA-4, and BTLA, expressed on T cells isolated from the BM of patients [4]. These molecules play an essential role in the loss of immune surveillance by regulating T cell activation and maintaining peripheral tolerance, and their significance for the development of solid tumors and hematologic malignancies has been well documented [5].

Impaired tumor immunity is suggested to be responsible for the very limited effectiveness of anti-myeloma immunotherapies in refractory MM [3]. Reversing tumor-mediated immune tolerance in MM seems to be a critical therapeutic goal in the development or optimization of new immunotherapeutic strategies. The introduction of inhibitors targeting the immune checkpoints remarkably shifted the paradigm in the treatment of solid tumors and hematologic malignancies with impressive single-agent responses for PD-1/PD-L1 axis inhibitors in Hodgkin’s lymphoma [6,7,8,9,10]. However, unlike solid-tumor patients, only a minor subset of MM patients treated with checkpoint inhibitors have been shown to derive clinical benefits, primarily after combined therapy, thus suggesting differential participation of inhibitory receptors or different categories of inhibitory pathways involved in tumor immunity [11]; in fact, it has recently been reported that T cells in MM patients display features of immunosenescence and exhaustion, and, notably, these dysfunctional states may coexist in time [5,12,13]. Although both senescence and the exhaustion of T cells are associated with compromised immune responses, they substantially differ in their phenotypic and functional characteristics, as well as underlying mechanisms [14]. Available data demonstrate that immunosenescence is caused by intrinsic signals induced by DNA damage or other stresses and can be reversed pharmacologically, while exhaustion is a consequence of ligation of inhibitory receptors and is reversible upon external receptor blockade [15]. Therefore, it is crucial to resolve immunosuppressive mechanisms by identifying the dominant inhibitory pathway in MM patients to achieve their effective control with therapeutic interventions.

Herein, we extended and completed our preliminary data to explore mechanisms underlying the systemic CD4 T cell-related immunosuppression according to the disease course to identify a target group suitable for therapeutic use of immune checkpoint inhibitors [16]. Therefore, we aimed to examine phenotypic and functional characteristics of CD4 T cells assigned to cell senescence or exhaustion, considering PD-1, CTLA-4, and BTLA checkpoint expression, as well as secretory effector function, including capacity for IL-17 and IFN-γ production. We assessed CD4 T cells from peripheral blood (PB) of active MM patients at disease onset and relapse, as well as healthy age-matched donors. Our study demonstrated that CD4 T cell senescence (associated with defective checkpoint expression in MM [13]) might play a role in supporting myeloma growth, whereas T cell exhaustion (characterized by recovery of checkpoint expression) is a predominant dysfunctional state at disease relapse, which may affect the clinical response to therapeutic checkpoint inhibitors in MM.

## 2. Results

### 2.1. CD4 T Cells from PB of MM Patients Are Maximally Stimulated In Vivo and Possess Strong Potential for Inhibition of the Immune Response

Having demonstrated that immune checkpoints function as negative feedback to regulate the ongoing immune responses and their dysregulated expression may be a consequence of altered in vivo stimulation [17], we analyzed the state of systemic activation and the capacity for re-stimulation of MM CD4 T cells.

While we noted an increased proportion of CD4^+^CD69^+^ T cells in the PB of all patients (as shown in Table 1), a statistically significant difference was found only between the RRMM group and healthy controls (*p* = 0.027); in the newly diagnosed (NDMM) group, the increase in CD69^+^ cell expansion was of borderline significance (*p* = 0.06). As demonstrated in Table 2, the median fluorescence intensity of CD69 was the highest in CD4 T cells from RRMM patients, but it remained at a statistically similar level compared to corresponding healthy cells. In contrast, CD4 T cells from NDMM patients exhibited markedly lower amounts of CD69 than those from the controls (*p* = 0.017), which did not differ significantly in comparison to the CD69 levels found in the RRMM group. The in vitro re-stimulation revealed that patients’ cultured CD4 T cells from both studied groups exhibited a significantly lower proportion of CD69^+^ cells than the corresponding healthy cells (44.29% (26.22%–54.72%) vs. 55.73% (53.00%–73.16%), *p* = 0.044) under the same stimulation conditions.

This part of the data shows that PB CD4 T cells in MM were maximally activated in vivo, but hypo-responsive and failed to respond to polyclonal re-stimulation. Among patients, a lower level of systemic CD4 T cell activation was observed at MM diagnosis.

### 2.2. Expression of Immune Checkpoints in Myeloma CD4 T Cell Subsets Is Clearly Impaired, Especially in Newly Diagnosed Patients

It is well established that immune checkpoint receptors play an essential role in immune surveillance and tumor immunity by inhibiting T-cell immune responses [5]. We and others have previously demonstrated the altered expression of inhibitory receptors CTLA-4, PD-1, and BTLA in tumors [4,18,19,20,21,22,23,24,25,26]. As recent clinical trials with administration of the immune checkpoint inhibitors in MM showed real disappointment, we aimed to verify whether the onset and/or exacerbation of MM is accompanied by alterations in the immune checkpoints’ expression, thereby affecting their usefulness as targets for therapeutic inhibitors. Therefore, we assessed PD-1, BTLA, and CTLA-4 checkpoint expression in PB CD4^+^ T cell subsets in MM patients both at disease diagnosis and relapse.

**Figure 1:**
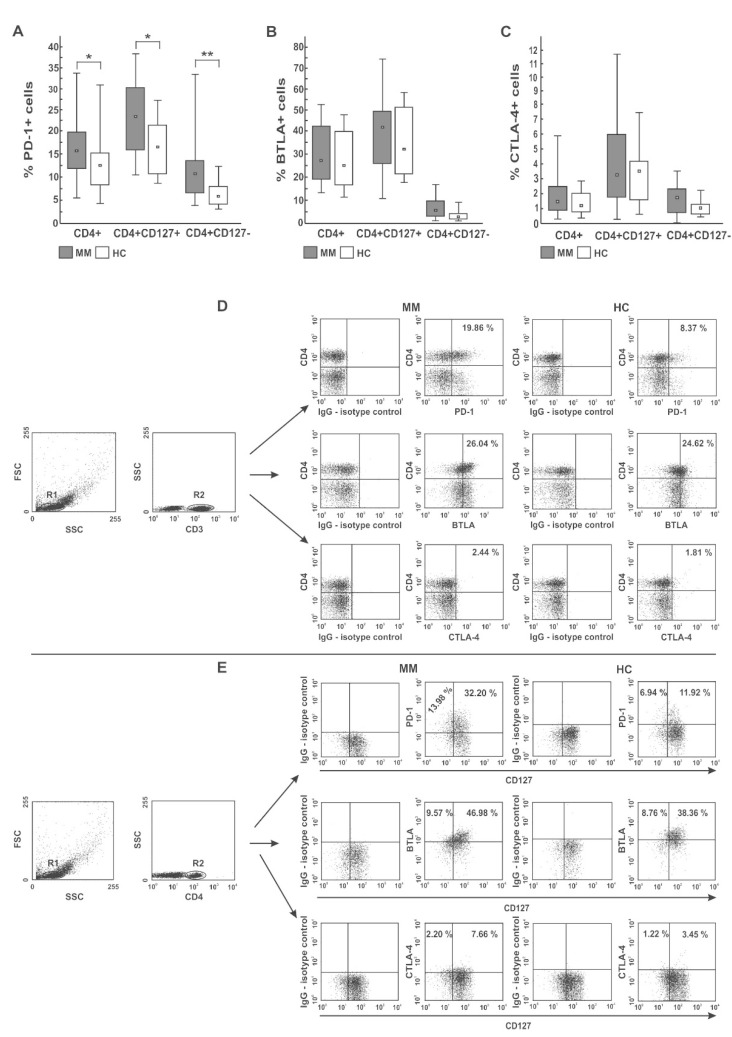
Distribution of PD-1 (**A**), BTLA (**B**), and CTLA-4 (**C**) immune checkpoints in different PB CD4 T-cell subsets. Among studied inhibitors, significant differences between myeloma patients (MM) and healthy controls (HC) were found in PD-1 expression only (*p* < 0.05). The frequency of BTLA^+^ and CTLA-4^+^ cells did not significantly differ between MM and HC (*p* > 0.05). Boxes and whiskers 25th–75th interquartile range and minimum-maximum, respectively; the median is the central square in each box. ** represents *p* < 0.01 and * represents *p* < 0.05. (**D**,**E**) Representative dot plots show PD-1, BTLA, and CTLA-4 expression in PB CD4 T cells. Numbers on dot plots represent the frequency of PD-1^+^, BTLA^+^, or CTLA-4^+^ cells within the examined subsets. The percentages of cells expressing the immune checkpoint receptors were determined using isotype control IgG. The statistical analysis was performed using the Mann-Whitney U-test.

As shown in Figure 1A,D,E, and Table 1, a comparison with healthy controls demonstrated an increasing median proportion of CD4 T cells expressing PD-1 checkpoint in all MM patients regardless of cell subsets (both Teff and Treg cells defined as CD4^+^CD127^+^ and CD4^+^CD127^−^ T cells, respectively) (*p* < 0.05). Although the expansion of PD-1+ Teff cells was similar in all patients, Treg cells from RRMM patients expressed the PD-1 molecule on a significantly higher proportion of cells than in the NDMM group (*p* = 0.037).

A quantitative analysis of PD-1 expression showed its lower levels in NDMM patients compared with healthy controls (Table 2). Whereas PD-1 deficit was observed in the whole population of NDMM CD4 T cells, including both Teff and Treg subsets, its loss was more pronounced in Treg cells (*p* = 0.016); in Teff cells, the decrease of PD-1 was of borderline significance (*p* = 0.08). Likewise, in the RRMM patients, PD-1 expression was also defective (primarily in the Treg subset); however, its median values were statistically comparable to those observed in corresponding healthy cells. Similarly, the differences in PD-1 expression between patient groups, although apparent, were not statistically significant (Table 2).

As demonstrated in Figure 1B,D,E, and Table 1, regarding BTLA expression, we found no significant differences in the percentages of BTLA^+^ cells within CD4 T cells and their subsets (both Teff and Treg cells) between patients and controls, except for the higher proportion of CD4^+^ and Treg cells co-expressing BTLA in the NDMM and RRMM groups, respectively (*p* = 0.018 and *p* = 0.01, respectively). In addition, compared with healthy cells, a decrease in the MFI values of BTLA in the MM CD4 T cells, more profound in NDMM patients, was observed (*p* < 0.002). BTLA levels in the Treg subset in NDMM patients were also lower than those observed in RRMM patients (*p* = 0.001) (Table 2).

Likewise, we found no significant differences in the proportion of CTLA-4 expressing cells within the examined subsets between participants studied, except for the higher abundance of CTLA-4^+^ Treg cells in RRMM patients compared with healthy controls (*p* = 0.031) (Table 1). Remarkably, its quantitative estimation showed that the only group exhibiting substantially down-regulated levels of CTLA-4 on both Teff and Treg cells was the NDMM patient group (*p* ≤ 0.008 and *p* ≤ 0.005, respectively) contrasting of the normal levels in corresponding cells from the RRMM and healthy groups (Table 2).

Taken together, these data clearly show that PD-1 is the only T cell inhibitory receptor widely distributed within PB CD4 T subsets in patients at every stage of MM and increasing within Treg population during disease progression. Nonetheless, myeloma CD4 T cells had significantly defective levels of all studied checkpoints, primarily at myeloma diagnosis, which may be insufficient for appropriate blockade with therapeutic inhibitors.

### 2.3. Dysfunctional Characteristics of PB CD4 T Cells Depend on Myeloma Stage

As altered expression of immune checkpoints is one of the features of cell senescence or exhaustion observed in MM [5,12,13], we wanted to assess whether it corresponds with the other dysfunctional characteristics of these two states, i.e., aberrant and opposed capacity for inflammatory IFN-γ and IL-17 cytokine secretion [27,28].

**Figure 2:**
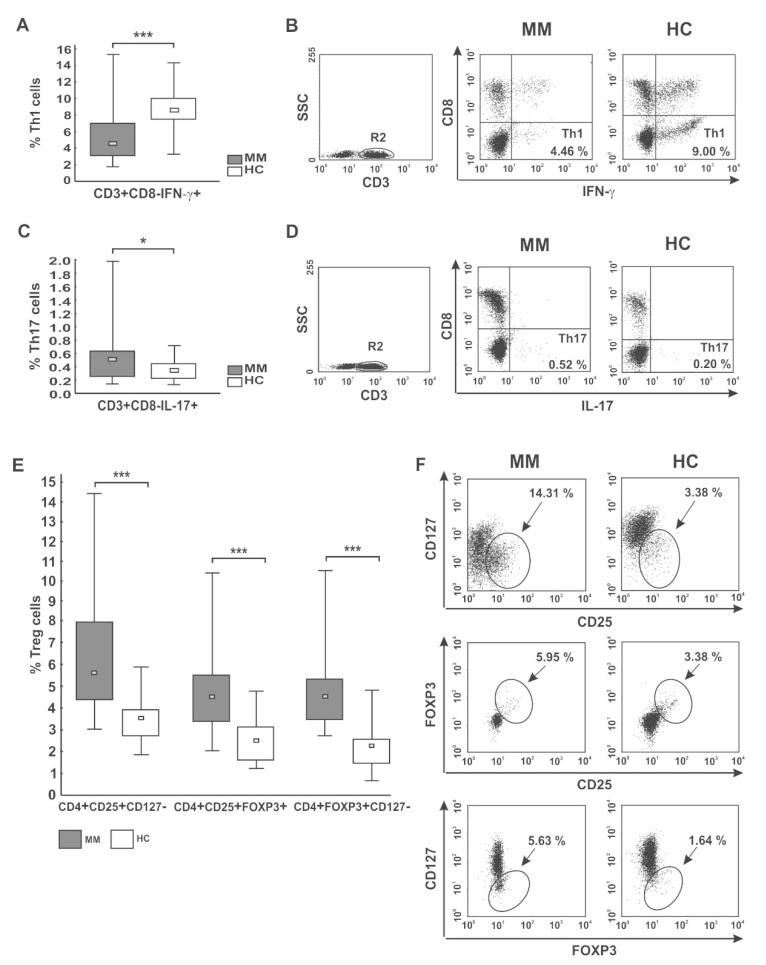
CD4 T cell capacity for cytokine secretion (IFN-γ, IL-17) and peripheral distribution of Treg cells. (**A**,**B**) IFN-γ- (Th1) and (**C**,**D**) IL-17-secreting cells (Th17) were phenotyped by flow cytometry as CD3^+^CD8^−^IFN-γ^+^ and CD3^+^CD8^−^IL-17^+^ cells, respectively. (**E**,**F**) Treg cells were identified as the following subsets: CD4^+^CD25^+^CD127^−^, CD4^+^CD25^+^FOXP3^+^, and CD4^+^FOXP3^+^CD127^−^ cells. Boxes and whiskers 25th–75th interquartile range and minimum-maximum, respectively; the median is the central square in each box. *** represents *p* < 0.001 and * represents *p* < 0.05. Numbers on dot plots represent the percentage of Th1, Th17, and Treg cells within PBMCs in MM patients and healthy subjects (HC). Significant decreases in PB Th1 and increases in both Th17 and Treg cells among patients were found in all analyses using the Mann-Whitney U-test.

**Figure 3:**
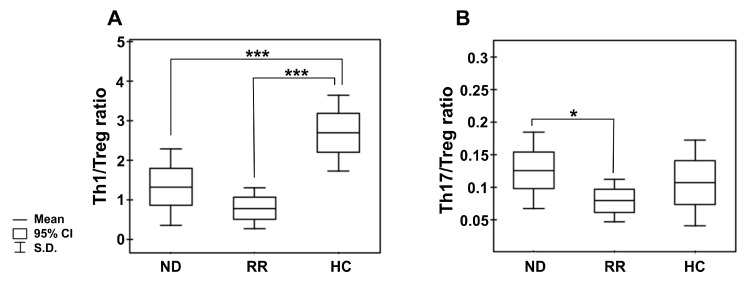
Peripheral blood Th1/Treg and Th17/Treg cell ratios in the different clinical phases of MM and healthy controls. (**A**) shows the ratio of the numbers of circulating IFN-secreting CD4 T cells (Th1) to regulatory CD4^+^CD25+CD127^−^ T cells (Treg); (**B**) shows the ratio of circulating IL-17-secreting CD4 T cells (Th17) to regulatory CD4^+^CD25^+^CD127^−^ T cells. Boxes and whiskers show confidence interval and standard deviation (S.D.) respectively; the mean is the central square in each box. *** represents *p* < 0.001 and * represents *p* < 0.05.

In the pooled MM group, we observed significantly diminished proportions of the CD4 T cells with capacity for IFN-γ secretion (Th1 subset) compared with healthy donors (*p* < 0.001) (as shown in Figure 2A,B, and Table 3). Moreover, we found markedly lower values of IFN-γ fluorescence intensity in the CD4 T cells than those seen in controls (31.86 (20.72–37.10) vs. 58.79 (36.41–69.87), *p* = 0.01). Although we did not find any significant differences in the abundance of Th1 cells in PB between patients regarding treatment state or ISS stage, a pronounced deficit was observed in the RRMM group and patients with higher tumor stage (Table 3 and Table S1). Likewise, while a substantial decline in the Th1/Treg cell ratio was observed in both groups of patients irrespective of MM phase (*p* = 0.00007 for NDMM patients, *p* = 0.00003 for RRMM patients), and patients at MM relapse exhibited the lowest Th1/Treg rate (Figure 3A).

Additionally, we assessed the level of PB CD4^+^ T cells capable of inflammatory IL-17 cytokine synthesis (Th17 cells). In patients, the frequencies of Th17 cells were significantly higher than in controls (*p* < 0.05) (Figure 2C,D), especially those at ISS stage I/II (Table S1). Nonetheless, the MFI values of IL-17 in the Th17 subpopulation were comparable to those observed in controls (23.39 (13.14–39.00) vs. 21.98 (16.37–52.11), *p* > 0.05, respectively). Although no significant difference in Th17 cell levels between patient groups was found (Table 3), we clearly observed that NDMM patients exhibited a markedly increased Th17/Treg ratio compared with those with RRMM (*p* = 0.047), as shown in Figure 3B. While the Th17/Treg ratio in RRMM was the lowest, it did not significantly differ to that observed in healthy controls.

This part of the data demonstrates that CD4 T cells from MM patients are functionally impaired but secrete more inflammatory cytokines during disease development than those at myeloma progression, which may imply different functional characteristics corresponding with, respectively, senescence or exhaustion depending on disease stage.

### 2.4. Expansion of PB Treg Cells Is Most Pronounced at Less Advanced MM, Which May Create Conditions Promoting Disease Development

Having ascertained that Treg cells might be involved in T cell senescence during tumor induction [29,30], we evaluated the abundance of PB Treg cells in the different clinical phases of MM. We determined the following Treg cell subsets: CD4^+^CD25^+^CD127^−^, CD4^+^CD25^+^FOXP3^+^, and CD4^+^FOXP3^+^CD127^−^ cells.

The median percentages of all studied Treg subtypes were significantly higher at every clinical stage of MM compared with controls (*p* ≤ 0.004) (Table 3; Figure 2E,F). Our cohort of active MM patients (both NDMM and RRMM) exhibited statistically comparable proportions of Treg cells; however, the CD4^+^CD25^+^CD127^−^ Treg subset was the only regulatory cell population found to tend to increase after therapy (*p* = 0.076). We also surprisingly noted that Treg levels were higher at stage I/II compared with the values observed at stage III, and the differences reached statistical significance for CD4^+^CD25^+^FOXP3^+^ and CD4^+^FOXP3^+^CD127^−^ subsets (both *p* = 0.02) (Table S1). Furthermore, among examined Treg subtypes, the abundance of CD127^−^ Treg cells (both CD4^+^CD25^+^CD127^−^ and CD4^+^FOXP3^+^CD127^−^ phenotypes) negatively correlated with ISS stage (*r* = −0.24, *p* = 0.04 and *r* = −0.49, *p* = 0.006, respectively) (Figure S1).

This part of our data clearly shows an increase in the circulating Treg cell compartment irrespective of treatment state, although more pronounced at less-advanced stages of myeloma. PB Treg enrichment observed at tumor induction may create conditions supporting CD4 T cell senescence-mediated systemic immune suppression.

### 2.5. Markers of T Cell Exhaustion Are Associated with Adverse MM Clinical Behavior

Since the impact of checkpoints’ up-regulated expression on the clinical outcome of neoplastic diseases has been demonstrated [4,18,19,20,21,22,23,24,25], we wanted to find out whether T cell inhibitors might be associated with clinical characteristics of MM as well. The associations between immune characteristics and both MM clinical variables and patient survival are summarized in Table 4 and Table 5, respectively.

As shown in Table 4, the patients with adverse clinical features, such as higher levels of β2-microglobulin (β2M) (≥ 3.5 mg/dL), IgA myeloma subtype, ISS stage > 2, and lower albumin level (≤ 3.5 g/dL) had higher expression of PD-1 checkpoint (*p* = 0.06, *p* = 0.01, *p* = 0.02, and *p* = 0.05, respectively), while patients with hypercalcemia (≥ 10 mg/dL of calcium) exhibited elevated levels of CTLA-4 on the pooled CD4 T cells and their subpopulations studied (Teff and Treg) (*p* = 0.02, *p* = 0.0006, and *p* = 0.002, respectively). Additionally, we noted an association between anemia (Hb ≤ 12 g/dL) and higher concentrations of β2M with increased frequencies of CD4^+^CD69^+^ T cells (*p* = 0.08 and *p* = 0.02, respectively).

Next, we aimed to evaluate whether any of the immune checkpoints associated with an unfavorable clinical course of MM might possess prognostic significance for overall survival (OS). We stratified the results obtained for low and high expression of each immune checkpoint according to the median split. Similar analysis was performed regarding clinicopathological variables known to be involved in MM progression and prognosis. The median follow-up of our cohort of MM patients was 27 months (range: 0–86 months).

Regarding clinical characteristics (as illustrated in Figure S2), a log-rank test showed that high β2M (Figure S2A), low albumin (Figure S2B), ISS stage > 2 (Figure S2C), and to a lesser extent anemia (Figure S2D), high creatinine levels (Figure S2E), and older age (Figure S2F) predicted shorter OS of patients (*p* = 0.0004, *p* = 0.003, *p* = 0.01, *p* = 0.06, *p* = 0.08, *p* = 0.09, respectively). There was no significant correlation between myeloma isotype, serum calcium concentration, circulating plasmocytes, or lactate dehydrogenase (LDH) level and patient OS (Figure S3).

Among immune features studied, only increased frequencies of both CD69^+^ (Figure S2G) and PD-1^+^ CD4 Teff cells (Figure S2H) predicted with borderline significance shortened patient OS (both *p* = 0.06); we observed that the intensity of PD-1 expression in CD4 T cells may have a minor effect on patient survival (*p* = 0.14) (Figure S2I).

In univariate Cox analyses (Table 5), clinical variables including low albumin, high creatinine and β2M levels, and anemia correlated with worse OS (*p* = 0.005, *p* = 0.04, *p* = 0.05, and *p* = 0.08, respectively); ISS stage > 2 and older age were also shown to associate with shortened OS to some extent (*p* = 0.11 and *p* = 0.10, respectively). In turn, no correlation was observed between patient OS and serum calcium level, LDH, and platelet and plasmocyte counts. Among the immune parameters studied, only the percentage of PD-1^+^ CD4 Teff cells was found to tend to slightly increase the risk of death (*p* = 0.10).

A multivariate Cox regression model was built including clinical prognostic factors and frequency of PD-1^+^ CD4 Teff cells and CTLA-4 level in CD4^+^ T cells (reaching *p* values ≤ 0.11 in univariate analysis). This multivariate analysis allowed the independent prognostic value for OS to be retained only for albumin level, anemia, and age (*p* = 0.001, *p* = 0.05, and *p* = 0.05, respectively) (Table 5).

Taken together, these data suggest that no immune feature could be added to the clinical scoring system in MM; however, CD4 T cells with predominance of the activated and exhausted phenotype are involved in adverse clinical behavior.

## 3. Materials and Methods

### 3.1. Samples and Patient Characteristics

The study group of patients consisted of a total of 40 active myeloma patients (26 newly diagnosed and 14 relapsed/refractory (RR)) (21 female). Patients were recruited in the Department of Hematology and Bone Marrow Transplantation at Wroclaw Medical University and the Department of Hematooncology at the Provincial Hospital in Opole, and diagnosed based on criteria from the International Myeloma Working Group (IMWG) [31]. The disease stage was determined according to the International Staging System (ISS) at the study entry [32]. Relapsed/refractory MM patients (RRMM) were treated with chemotherapy, immunomodulatory drugs, and proteasome inhibitor; no patient enrolled in the study received prior treatment with stem cell transplantation (SCT) or immune checkpoint inhibitors. The baseline characteristics of the patients are shown in Table 6. The control population comprised 20 healthy individuals matched for age and sex; they had been without any treatment affecting the immune system for 6 months before entering the study. Patients with simultaneous active or chronic infection, diabetes, autoimmune disease, or with a history of other malignancies or connective tissue diseases were excluded from the study. Blood samples from all participants were collected after informed consent in accordance with the Declaration of Helsinki and approval by the Institutional Local Research Bioethics Committee at Wroclaw Medical University.

### 3.2. Cell Isolation from Peripheral Blood

Peripheral blood mononuclear cells (PBMCs) were isolated by Lymphoflot (Bio-Rad Medical Diagnostics GmbH, Dreieich, Germany) density gradient centrifugation from venous blood samples of patients with MM and healthy donors, and then cryopreserved. Recovery rates from frozen T cells were above 85%.

### 3.3. Secretory Effector Function, Immunofluorescence Staining, and Flow Cytometric Analysis

Peripheral blood mononuclear cells (PBMCs) were stained with several combinations of anti-human fluorochrome-conjugated monoclonal antibodies (mAbs) for multi-color analysis. For assessment of the dominant inhibitory pathway in the pooled CD4^+^ T cells as well as their subsets (both Treg and Teff cells, phenotyped according to Liu et al. [33] as CD4^+^CD127^−^ and CD4^+^CD127^+^ cells, respectively), surface staining of CD4, CD69, BTLA, PD-1, CTLA-4, and CD127 was performed by standard protocols. The following mAbs were used in this procedure: CD3-FITC (Pharmingen, San Diego, CA, USA), CD4-PerCP (Pharmingen, USA), BTLA-PE (Becton Dickinson, Biosciences, San Diego, CA, USA), PD-1-PE (Pharmingen, San Diego, CA, USA), CTLA-4-PE (Pharmingen, San Diego, CA, USA), CD69-PE (Pharmingen, San Diego, CA, USA), and CD127-FITC (Pharmingen, San Diego, CA, USA).

For analysis of the regulatory T cell (Treg) subpopulations phenotyped as CD4^+^CD25^+^CD127^−^, CD4^+^CD25^+^FOXP3^+^, and CD4^+^CD127^−^FOXP3^+^ cells, PBMCs were aliquoted into tubes directly after isolation for further staining with the following mAbs: anti-CD4-PerCP (BD Biosciences, San Diego, CA, USA), anti-CD25-FITC (BD Biosciences, San Diego, CA, USA), and anti-CD127-PE (BioLegend, San Diego, CA, USA), respectively. For intracellular staining, the cells were then fixed and permeabilized with the Fixation/Permeabilization Buffer Set (eBioscience, San Diego, CA, USA) according to the manufacturer’s instructions with subsequent incubation with anti-human FOXP3-PE (BD Biosciences, San Diego, CA, USA) mAbs for 30 min at room temperature in the dark.

For assessment of the secretory effector function of CD4^+^ T cells, staining of intracellular cytokines IL-17 and IFN-γ was performed. Percentages of cytokine-producing T cells (Th1 and Th17) were calculated after stimulation of refrozen PBMCs with 25 ng/mL phorbol 12-myristate 23-acetate (PMA, Sigma-Aldrich, Merck KGaA, Darmstadt, Germany) and 1 µg/mL ionomycin (Ion) (Sigma-Aldrich, Merck KGaA, Darmstadt, Germany)h) in the presence of 10 µg/mL brefeldin A (BFA, protein transport inhibitor) (Sigma-Aldrich, Merck KGaA, Darmstadt, Germany) for 4 h at 37 °C in a humidified atmosphere containing 5% CO_2_. Because incubation with PMA triggers internalization and degradation of the CD4 molecule, which would affect the identification of Th1 (phenotyped as CD4^+^IFN-γ^+^) and Th17 cells (characterized as CD4^+^IL-17^+^) [34], we decided to identify both subpopulations as CD3^+^CD8^−^IFN-γ^+^ and CD3^+^CD8^−^IL-17^+^, respectively. Directly after PMA+Ion stimulation, PBMCs were surface-stained with anti-CD3-PerCP (BD Biosciences, San Diego, CA, USA) and anti-CD8-PE mAbs (BD Biosciences, San Diego, CA, USA). Then, after fixation and permeabilization with the Fixation/Permeabilization Buffer Set (eBioscience, San Diego, CA, USA), the cells were incubated with anti-IFN-γ-FITC (BD Biosciences, San Diego, CA, USA) or anti-IL-17-FITC (BioLegend, San Diego, CA, USA) mAbs for 30 min at room temperature in the dark.

Directly after immunostaining, the cells were washed and analyzed by flow cytometry using a FACScan cytometer (Becton Dickinson, San Diego, CA, USA) equipped with Cell Quest software (BD Biosciences, San Diego, CA, USA). Appropriate fluorochrome-labeled isotypic controls were used to confirm expression specificity and for gate settings in each case. A total of 100,000 events were recorded for each sample before any electronic gate setting. Data were analyzed by Cell Quest software.

The results were expressed as the proportions of CD3^+^CD4^+^ (CD4 T cells), as well as CD4^+^CD127^−^ and CD4^+^CD127^+^ cells (Treg and Teff, respectively) co-expressing inhibitory receptors BTLA, PD-1, or CTLA-4. The percentages of CD3^+^CD8^−^ co-expressing IFN-γ (Th1 subset) or secreting IL-17 (Th17 subset) were also examined. In addition, we studied the frequencies of CD4^+^CD25^+^ cells with the presence of the FOXP3 transcription factor and/or without or with low expression of CD127 antigen, thus defining the different subsets of Tregs. In order to demonstrate quantitative expression of studied molecules at the single-cell level, the results are shown as the mean fluorescence intensity (MFI) values and expressed in arbitrary units (AU).

### 3.4. Statistical Analysis

Statistical analysis was performed using the package Statistica 7.1 (TIBCO Software Inc., Palo Alto, CA, USA) and GraphPad Prism 8.01 (GraphPad Software, San Diego, CA, USA). Clinical parameters were presented as absolute numbers and percentages for frequencies. For all other analyzed variables, the median values and 25th and 75th interquartile ranges (IQ ranges) were calculated. As collected data were not normally distributed and/or had heterogeneous variances, differences between examined groups were evaluated using nonparametric tests for paired (Friedman, Wilcoxon) and unpaired (Kruskal-Wallis, Mann-Whitney U) data. The relationship between the ISS stage and other analyzed variables was evaluated by Kendall’s tau coefficient analysis. Kaplan-Meier curves were generated to present the survival time of the two groups, and the differences were assessed by the log-rank test. Multivariate analyses were performed with the Cox proportional hazards model by including all statistically significant covariates from univariate Cox models. A *p* value ≤ 0.05 was considered significant.

## 4. Discussion

The results of the present study clearly support an important role of the immune checkpoints in the development of systemic T cell immune dysregulation in active myeloma. Our study strengthens the suggestion that myeloma growth disrupts both the qualitative and quantitative expression of immune checkpoints in the PB CD4^+^ T cell subsets, which may complicate the clinical response to therapeutic checkpoint inhibitors. Here, we observed that among the studied immune checkpoints, PD-1 was the only inhibitory receptor found in higher proportions of PB Teff and Treg cells and correlated with adverse MM clinical outcomes. This observation is consistent with the report of Rosenblatt et al. [4], who observed an increased frequency of PB CD4^+^PD-1+ T cells in myeloma patients with advanced active disease as a result of chronic antigen stimulation, thus contributing to tumor-induced suppression of T cell responses. A reduction of PD-1^+^ T cell frequency in patients who achieve a minimal disease state following chemotherapy strongly supports an association of PD-1 expression with exposure to the tumor antigens and stimulation in vivo. Consistent with tumor antigen exposure, we observed increased frequencies of in vivo-stimulated MM CD4^+^CD69^+^ T cells, although exhibiting lower potential to respond to further in vitro polyclonal stimulation, thus indicating a dysfunctional phenotype of PB CD4^+^ T cells. This notion together with the increased expression of PD-1 within the CD4 T cell subset and the severely impaired Th1 response seems to reflect an in vivo-stimulated and most likely exhausted phenotype of CD4^+^ T cells in our cohort of active patients, especially those with relapsed MM. In fact, increased expression of inhibitory receptors PD-1, CTLA, LAG-3, and TIM-3, together with defective effector functions, is regarded as a hallmark of T cell exhaustion [25,26,28]. The influence of MM therapy on quantitative and functional characteristics of circulating CD4 T cells has consistently been reported by Batorov et al. [35]. While it has been found that in the course of MM T cell exhaustion occurs predominantly in the myeloma BM, PB T cells also exhibit an abrogated function, albeit to a minor extent [5,25]. Our study suggests that MM relapse (and treatment refractoriness) is associated with an increasing population of activated and exhausted PB CD4 T cells, which may clearly affect the clinical outcome, as shown by the correlation with hypercalcemia, high β2M levels, low albumin levels, a possible association with anemia, and shortened survival.

Remarkably, we also observed that systemic checkpoints’ expression examined at a single-cell level on the different types of CD4 T cells was clearly impaired mainly at diagnosis of MM. This notion is in line with recent studies by Suen et al. [12,13], who reported decreased levels of PD-1 and CTLA-4 on clonal T cells in MM patients as a feature of telomere-independent immunosenescence rather than exhaustion. Likewise, we found that the CD4 T cell compartment in patients with disease onset was characterized by relatively higher capacity for the secretion of inflammatory IL-17 and IFN-γ cytokines compared with patients with relapsed and advanced disease, which may be a characteristic of the senescent-associated secretory effector phenotype (SASP) [27]. T-cell senescence is believed to be an alternative mechanism of immune evasion utilized by malignant cells for tumor development [36,37,38], as senescent T cells were shown to be an important source of immunosuppressive cytokines, such as IL-10 and TGF-β [30]. It is also postulated that Treg cells are involved in conversion of normal T cells into senescent cells [29,30]. Our finding of a negative correlation of enriched PB Treg cells with MM stage may correspond with their role in systemic CD4 T cell senescence supporting myeloma growth. There is increasing evidence that senescence and exhaustion of CD4 T cells represent two different categories of inhibitory pathways leading to functional immune suppression [14]. Therefore, our study indicated that development and relapse of MM are likely related to dynamic changes in dysfunctional characteristics of PB CD4 T cells and confirmed recent data showing that immunomodulatory drugs and chemotherapy of MM are preferentially able to delete senescent T cells while retaining checkpoint inhibitory molecule expression [5]. Distinguishing between senescent and exhausted T cells, and targeting both types of cells in MM, may be of great clinical relevance, since reversion of these two dysfunctional states require different therapeutic approaches, among which checkpoint blockade has been reported to reverse only T cell exhaustion. We believe that an assessment of the level of immune checkpoints on T cell subsets may facilitate the identification of the predominant dysfunctional state of T cells in MM to improve therapeutic efficacy.

In accordance with the results of our quantitative analysis, Lee et al. [39] reported different expression levels of PD-1 regarding the clinical course of MM; patients in a refractory state exhibited markedly higher PD-1 amounts on T cells compared with those at diagnosis. Likewise, the CTLA-4 expression was also recently found to be lower and increasing with MM progression [39], an observation consistent with our finding of the significant increase in CTLA-4 fluorescence intensity on CD4 T cells (both Teff and Treg) to normal levels in patients with refractory disease. Although the majority of available data demonstrated an increase in the immune checkpoints’ expression in MM T cells, one should emphasize that they were based on qualitative assessment only [9,10,39,40]. A few recent reports [12,13,39] demonstrating the involvement of the quantitative alterations of immune checkpoints’ expression in pathogenesis and the clinical course of myeloma are similar to the results of our study, and point to the importance of their estimation at the quantitative level as well. The inappropriate checkpoint levels in MM T cell subsets observed in our study, primarily in newly diagnosed patients, might explain the sub-optimal clinical responses in clinical studies using checkpoint inhibitors and the real disappointment with this therapeutic modality in MM [41,42]. This is in sharp contrast to the impressive response to blockade of CTLA-4, PD-1, and PD-L1 seen in a broad variety of cancers of different origin [43], and strengthens the suggestion of the requirement for a relevant expression level of checkpoints on T cells. Consistently, we previously reported that CTLA-4 blocking antibody might be a beneficial form of immunotherapy for a subset of chronic lymphocytic leukemia (CLL) patients depending on the level of CTLA-4 expression on leukemic cells [23].

The reason for the down-regulation of the checkpoints’ expression level in CD4 T cells in a proportion of MM patients is still unresolved, although in light of the higher CD69 values seen in our study, insufficient in vivo stimulation of MM T cells should be excluded. The influence of the transcription factors (such as Blimp or T-bet) that have been demonstrated to control the checkpoint expression might also be considered [44,45]. In addition, recent research performed on MM, including from our group, demonstrated that genetic variations of genes encoding the immune checkpoints, primarily PD-1 and CTLA-4, may affect their protein expression level as well [46,47,48]. While Katsumoto et al. [45] stated that PD-1 high-expression haplotype is implicated in susceptibility to MM, we previously reported that polymorphisms in the *CTLA-4* gene associated with lower CTLA-4 protein expression significantly increase the risk of developing MM in the Polish population [47]. Similarly, Zheng et al. [48] found that an (AT)n microsatellite polymorphism within the 3ʹ-untranslated region (UTR) of exon 3 of the *CTLA*-4 gene might represent a susceptibility locus for MM, as the increased frequencies of the alleles containing extended AT repeats seen in MM patients are associated with lower CTLA-4 mRNA stability and protein expression.

Herein, we confirmed the independent prognostic value of age, albumin, hemoglobin, and β2M levels, thus indicating the clinical representativeness of patients enrolled in the study. Yet, the only immune characteristic found to predict a poor clinical outcome in MM was the PD-1 checkpoint expressed on PB CD4 Teff cells; patients with higher expression of PD-1 had an unfavorable clinical course and tended to live shorter. Our observation is in line with the report by Alrasheed et al. [49], who reported independent prognostic significance of high abundance of PD-1^+^CD4 Teff cells in the prediction of early relapse of MM. The relatively small cohort of patients included in the current analysis might weaken the significance of Cox regression analysis with regards to examined immune parameters. Likewise, clinical studies showing that among therapeutic checkpoint inhibitors, only the anti-PD-1 antibody revealed a clinical response, although sub-optimal, in a proportion of MM patients when administered in a combined therapy only, might strengthen the possible contribution of PD-1 to prognosis in MM [50,51]. Further studies including larger cohorts of MM cases are required to verify our findings.

It is worth noting that among the immune checkpoints studied, PD-1 expression was found to be the most deregulated, when considering co-existence of qualitative PD-1 overexpression at every stage of MM with quantitative impairment of PD-1 at disease development. While CTLA-4 expression was found to be associated with hypercalcemia, our observation of the PD-1 expression increasing with several other features of adverse clinical courses, such as advanced ISS stage, higher level of β2M, and decreased albumin levels and anemia, emphasizes a superior role of the PD-1 inhibitory receptor in the development of systemic immune suppression and myeloma progression. It has been reported that the widespread expression of PD-1L on neoplastic plasma cells and dendritic cells (DC) facilitates interaction with PD-1 on the marrow-infiltrating lymphocytes (MILs), and strongly restricts anti-tumor T cell responses within the BM microenvironment, thereby allowing for the tumor escape [52,53]. This is in accordance with the demonstration that PD-1 enhances regulatory properties in Treg cells and inhibition of anti-tumor activity of CD4 Teff cells in MM, indicating a role of PD-1 in the MM clinical outcome [49]. In fact, it has been found that the PD-1^+^ Treg subset is the main population participating in immune deficiency during tumor progression [24,52]. Additionally, a role of PD-1 in conversion of Th1 into Treg cells was recently demonstrated [54], thus emphasizing the significance of the PD-1 checkpoint for a shift of the immune balance towards immune suppression due to a decline in the Th1/Treg ratio [49]. Our observation on the enrichment of PB Treg cells in all MM patients is in line with a role of PD-1 in Treg expansion. Although we observed a decrease in FOXP3^+^Tregs at stage III, we noted that their values still remained increased in the periphery irrespective of tumor stage. At this point of the study, we cannot completely explain the FOXP3+Treg decrease in the most advanced MM (ISS stage III), but our findings confirmed the similar former observation [55]. Infiltration of the BM by Tregs should be taken into consideration, since these cells have been shown in MM to acquire chemokine receptors promoting trafficking to the tumor site. In fact, Tregs accumulate in the BM primarily in the most advanced disease, whereby they become capable of creating a highly immunosuppressive microenvironment supporting tumor growth [5,56,57].

The decrease in the PB CD4 T cell compartment secreting IFN-γ, a Th1 cytokine involved in tumor immunity, observed in our cohort of patients seems to reflect severe inhibition of anti-tumor effector functions of these cells in MM. The deficit in the Th1 type response observed in our study is likely associated with tumor progression, as we observed that patients with refractory advanced MM exhibited the lowest Th1 cell level and Th1/Treg ratio. The impact of the treatment-induced increase in PD-1 level on the compromised Th1/Treg ratio observed in the present study is consistent with recent observations [35] and may reflect the deterioration of T-cell-tumor immunity despite the treatment. Nonetheless, normalization of checkpoint levels on CD4 T cells in treated patients, despite development of refractoriness, appears to open an avenue for the reactivation of the immune responses after therapeutic use of checkpoint inhibitors in the combined modality. In conclusion, although the contribution of PD-1 to MM clinical outcomes is suggestive, our study clearly indicated that inappropriate expression of immune checkpoints (associated with the dysfunctionality of CD4 T cells and disease stage) might be responsible for the sub-optimal clinical response to checkpoint inhibitors in MM. Our data demonstrating defective levels of PD-1 and CTLA-4 within the CD4 T cell population in newly diagnosed patients suggest that immune checkpoints are not appropriate targets for therapeutic inhibitors at disease onset. This study also showed that chemo- and/or immunotherapy of MM, despite a risk of the development of refractoriness, is capable of reinforcing checkpoint expression and T cell reactivity of PB CD4 T cells, making them more attainable to therapeutic inhibitors in relapsed MM patients only.

## Figures and Tables

**Table 1:** Immune checkpoint and CD69 expression (%) in PB CD4 T cell subsets in different MM phases and healthy controls (HC).

T-Cell Subset (%)	At Diagnosis (NDMM) (*n* = 26)	At Relapse (RRMM) (*n* = 14)	HC (*n* = 20)	*p* Value
CD3^+^CD4^+^PD-1^+^	15.33 (12.62–18.52)	17.72 (11.75–19.86)	12.55 (8.37–15.29)	(a) ns (b) 0.02 (c) 0.04
CD4^+^CD127^+^PD-1^+^	23.53 (16.42–29.99)	25.00 (12.96–31.13)	16.87 (10.52–21.75)	(a) ns (b) 0.088 * (c) 0.090 *
CD4^+^CD127^−^PD-1^+^	9.70 (6.78–11.94)	14.79 (5.31–26.50)	6.14 (3.90–7.62)	(a) 0.037 (b) 0.041 (c) 0.0001
CD3^+^CD4^+^BTLA^+^	28.64 (20.04–43.21)	23.85 (15.06–30.73)	19.03 (13.25–37.52)	(a) ns (b) 0.018 (c) ns
CD4^+^CD127^+^BTLA^+^	38.87 (26.26–50.23)	40.30 (26.08–47.58)	23.62 (10.85–40.79)	(a) ns (b) ns (c) ns
CD4^+^CD127^−^BTLA^+^	3.98 (2.54–6.29)	7.70 (5.21–11.35)	2.47 (1.24–5.15)	(a) ns (b) ns (c) 0.010
CD3^+^CD4^+^CTLA-4^+^	1.91 (0.78–3.37)	1.28 (0.89–1.88)	1.27 (0.78–2.00)	(a) ns (b) ns (c) ns
CD4^+^CD127^+^CTLA4^+^	3.23 (2.21–7.66)	3.42 (1.68–4.36)	3.55 (1.64–4.22)	(a) ns (b) ns (c) ns
CD4^+^CD127^−^CTLA4^+^	1.93 (0.47–2.67)	1.68 (1.45–2.19)	1.15 (0.57–1.38)	(a) ns (b) ns (c) 0.031
CD3^+^CD4^+^CD69^+^	0.58 (0.23–1.09)	0.70 (0.36–0.95)	0.34 (0.22–0.44)	(a) ns (b) 0.060 * (c) 0.027

(a) NDMM vs. RRMM; (b) NDMM vs. HC; (c) RRMM vs. HC; *—trend; ns—not statistically significant. Differences in fluorescence intensity of immune checkpoints between examined groups were evaluated using nonparametric tests (Kruskal-Wallis and Mann-Whitney U-test).

**Table 2:** Immune checkpoint and CD69 expression (MFI, mean fluorescence intensity) in PB CD4T cells in different MM phases and healthy controls (HC).

Fluorescence Intensity	At Diagnosis (NDMM) (*n* = 26)	At Relapse (RRMM) (*n* = 14)	HC (*n* = 20)	*p* Value
PD-1 in CD4^+^	72.99 (67.04–86.64)	79.41 (56.39–111.33)	83.04 (71.73–105.09)	(a) ns (b) 0.063 * (c) ns
PD-1 in CD4^+^CD127^+^	67.19 (52.34–84.45)	85.34 (57.28–112.96)	90.27 (60.38–105.84)	(a) ns (b) 0.085 * (c) ns
PD-1 in CD4^+^CD127^−^	68.47 (54.77–93.55)	73.92 (59.29–113.76)	102.13 (79.79–127.27)	(a) ns (b) 0.016 (c) ns
BTLA in CD4^+^	169.60 (146.40–184.92)	184.12 (109.50–196.04)	215.18 (192.79–289.32)	(a) ns (b) 0.0002 (c) 0.002
BTLA in CD4^+^CD127^+^	150.75 (142.37–167.89)	186.19 (149.70–241.82)	240.20 (166.25–304.14)	(a) ns (b) 0.001 (c) ns
BTLA in CD4^+^CD127^−^	147.00 (120.49–168.15)	192.21 (154.61–235.12)	240.60 (167.13–292.31)	(a) 0.033 (b) 0.001 (c) ns
CTLA-4 in CD4^+^	44.95 (38.52–63.20)	76.21 (63.09–129.84)	65.40 (60.10–111.23)	(a) 0.002 (b) 0.005 (c) ns
CTLA-4 in CD4^+^CD127^+^	36.22 (33.40–41.87)	55.58 (49.57–95.97)	80.96 (51.93–90.08)	(a) 0.0002 (b) 0.0005 (c) ns
CTLA-4 in CD4^+^CD127^−^	34.13 (30.84–40.13)	53.69 (43.86–66.96)	62.97 (41.04–74.55)	(a) 0.008 (b) 0.0008 (c) ns
CD69 in CD4^+^	53.64 (47.87–58.82)	75.69 (49.52–94.75)	70.81 (55.55–123.46)	(a) ns (b) 0.017 (c) ns

(a) NDMM vs. RRMM; (b) NDMM vs. HC; (c) RRMM vs. HC; *—trend; ns—not statistically significant. Differences in the proportions of PD-1, BTLA, and CTLA-4 expressing CD4^+^ T cells between examined groups were evaluated using nonparametric tests (Kruskal-Wallis and Mann-Whitney U-test).

**Table 3:** Frequency of CD4 T cells secreting inflammatory cytokines and Treg cells in different MM phases and healthy controls (HC).

T-Cell Subset (%)	At Diagnosis (NDMM) (*n* = 26)	At Relapse (RRMM) (*n* = 14)	HC (*n* = 20)	*p* Value
CD3^+^CD8^−^IFN-γ^+^	4.79 (3.34–7.03)	3.91 (3.01–6.40)	9.00 (7.66–10.07)	(a) ns (b) 0.005 (c) 0.0002
CD3^+^CD8^−^IL-17^+^	0.51 (0.24–0.59)	0.56 (0.29–1.08)	0.32 (0.22–0.45)	(a) ns (b) 0.048 (c) 0.045
CD4^+^CD25^+^CD127^−^	5.02 (4.40–6.02)	6.61 (5.70–8.98)	3.69 (2.72–3.90)	(a) 0.076 * (b) 0.0001 (c) 0.0001
CD4^+^CD25^+^FOXP3^+^	4.30 (3.43–5.34)	4.58 (3.88–5.60)	2.51 (1.66–3.19)	(a) ns (b) 0.0001 (c) 0.001
CD4^+^ FOXP3^+^CD127^−^	4.52 (3.79–5.35)	4.35 (3.42–6.17)	2.34 (1.49–2.67)	(a) ns (b) 0.0001 (c) 0.001

(a) NDMM vs. RRMM; (b) NDMM vs. HC; (c) RRMM vs. HC; *—trend; ns—not statistically significant. Differences in Th1, Th17, and Treg cell subsets between examined groups were evaluated using nonparametric tests (Kruskal-Wallis and Mann-Whitney U-test).

**Table 4:** Association of immune features with clinical characteristics of multiple myeloma.

Cell Subsets	Clinical Features	Median (IQ Range)	*p* Value
CD4^+^CD127^+^PD-1^+^ (%)	<3.5 mg/L of β2M	18.66 (13.76–24.29)	
	≥3.5 mg/L of β2M	23.99 (16.42–32.32)	0.06 *
PD-1 in CD4^+^ (MFI)	≤3.5 g/dL of albumin	84.42 (72.42–129.93)	
	>3.5 g/dL of albumin	69.05 (55.08–94.03)	0.05
PD-1 in CD4^+^CD127^+^ (MFI)	IgA	107.59 (99.75–121.15)	
	IgG	63.85 (52.34–84.45)	0.01
PD-1 in CD4^+^CD127^−^ (MFI)	IgA	123.41 (104.10–149.45)	
	IgG	62.38 (54.77–85.70)	0.01
PD-1 in CD4^+^CD127^−^ (MFI)	Stage ≤ 2	59.49 (53.62–85.70)	
	Stage > 2	96.70 (69.07–113.76)	0.02
CTLA-4 in CD4^+^ (MFI)	<10 mg/dL of serum calcium	44.48 (39.87–62.12)	
	≥10 mg/dL of serum calcium	72.71 (45.56–98.68)	0.01
CTLA-4 in CD4^+^CD127^+^ (MFI)	<10 mg/dL of serum calcium	35.87 (32.94–39.74)	
	≥10 mg/dL of serum calcium	51.21 (44.45–72.31)	0.0006
CTLA-4 in CD4^+^CD127^−^ (MFI)	<10 mg/dL of serum calcium	33.79 (27.96–35.29)	
	≥10 mg/dL of serum calcium	53.69 (40.72–66.97)	0.002
CD4^+^CD69^+^ (%)	≤12 g/dL of hemoglobin	0.69 (0.56–1.09)	
	>12 g/dL of hemoglobin	0.37 (0.21–0.91)	0.08 *
CD4^+^CD69^+^ (%)	<3.5 mg/L of β2M	0.30 (0.19–0.68)	
	≥3.5 mg/L of β2M	0.69 (0.37–1.09)	0.02

MFI—mean fluorescence intensity; IQ—interquartile; *—trend.

**Table 5:** Univariate and multivariate Cox regression analysis.

Prognostic Factors	Univariate	Multivariate	
	*p* Value	HR (95% CI)	*p* Value
Age > 65 years	0.10 *	9.74 (0.94–100.33)	0.05
ISS stage > 2	0.11 *		
Albumin < 3.5 g/dL	0.005 *	0.04 (0.01–0.29)	0.001
β2-microglobulin ≥ 3.5 mg/L	0.05 *	21.65 (0.65–714.02)	0.08
Creatinine ≥ 2.0 mg/dL	0.04 *		
Serum calcium ≥ 10 mg/dL	0.91		
LDH > 190 U/L	0.96		
Hemoglobin ≤ 12 g/dL	0.08 *	0.16 (0.02–1.04)	0.05
Platelets < 100,000/mm^3^	0.43		
Plasmocytes > 5 %	0.69		
CD4^+^CD127^+^PD-1^+^ > median (%)	0.10 *		
PD-1 in CD4^+^ > median (MFI)	0.56		
PD-1 in CD4^+^CD127^+^ > median (MFI)	0.38		
PD-1 in CD4^+^CD127^−^ > median (MFI)	0.34		
CTLA-4 in CD4^+^ > median (MFI)	0.11 *		
CTLA-4 in CD4^+^CD127^+^ > median (MFI)	0.65		
CTLA-4 in CD4^+^CD127^−^ > median (MFI)	0.58		
CD4+CD69^+^ (%) > median (MFI)	0.20		

MFI—mean fluorescence intensity; HR—hazard ratio; CI—confidence interval; *—selected for multivariate analysis.

**Table 6:** Patient demographics and characteristics.

Characteristic	Newly Diagnosed (NDMM)	Relapsed/Refractory (RRMM)	Total
Number of patients, *n* (%)	26 (65%)	14 (35%)	40 (100%)
Sex (female)	17 (65.5%)	4 (28.5%)	21 (52.5 %)
Age of sampling (median, range)	66 (50–76)	72 (65–75)	69 (59–76)
ISS			
I	5 (19%)	1 (7%)	6 (15.0 %)
II	10 (38.5%)	6 (43%)	16 (40.0 %)
III	11 (42.5%)	7 (50%)	18 (45.0 %)
Myeloma isotype			
IgG	18 (69%)	9 (64%)	27 (67.5 %)
IgA	3 (11.5%)	3 (21.5%)	6 (15.0 %)
Light chain only	5 (19.5%)	2 (14.5%)	7 (17.5 %)
Type of Ig light chain (serum)			
Kappa	16 (61.5%)	7 (50%)	23 (57.5 %)
Lambda	9 (34.5%)	7 (50%)	16 (40.0 %)
None	1 (4%)	0 (0%)	1 (2.5 %)
Osteolytic bone lesion/s, *n* (%)	15 (60.0%)	13 (92.8%)	28 (70.0 %)
Laboratory values			
β2-microglobulin ≥ 3.5 mg/L	18 (69%)	9 (64%)	27 (67.5 %)
Creatinine ≥ 2.0 mg/dL	9 (34.5%)	4 (28.5%)	13 (32.5 %)
LDH > 190 U/L	6 (23%)	2 (14%)	8 (20.0 %)
Serum calcium ≥ 10 mg/dL	9 (34.5%)	11 (78.5%)	20 (50.0 %)
Hemoglobin ≤ 12 g/dL	23 (88.5%)	8 (57%)	31 (80.0 %)
Platelets < 100,000/mm^3^	2 (7.5%)	1 (7%)	3 (7.5 %)
Prior treatment			
1–3 therapy lines	0 (0%)	8 (57%)	8 (20.0 %)
≥4 therapy lines	0 (0%)	6 (43%)	6 (15.0 %)
BTZ-based therapy	0 (0%)	12 (85.5%)	12 (30.0 %)
IMiD therapy	0 (0%)	11 (78.5%)	11 (27.5 %)
No therapy	26 (100%)	0 (0%)	26 (65.0 %)

Abbreviations: BTZ, bortezomib; LDH, lactate dehydrogenases; ISS, International Staging System; IMiD, immunomodulatory drug; UNV, upper normal values.

## Data Availability

All data generated or analyzed during this study are available on request from the corresponding author.

## Supplementary Materials

The following are available online at https://www.mdpi.com/article/10.3390/ijms22179298/s1.

## References

[1] Kyle R.A., Rajkumar S.V. (2008). Criteria for diagnosis, staging, risk stratification and response assessment of multiple myeloma. Leukemia.

[2] Díaz-Tejedor A., Lorenzo-Mohamed M., Puig N., García-Sanz R., Mateos M.-V., Garayoa M., Paíno T. (2021). Immune System Alterations in Multiple Myeloma: Molecular Mechanisms and Therapeutic Strategies to Reverse Immunosuppression. Cancers.

[3] Tamura H. (2018). Immunopathogenesis and immunotherapy of multiple myeloma. Int. J. Hematol..

[4] Rosenblatt J., Glotzbecker B., Mills H., Vasir B., Tzachanis D., Levine J.D., Joyce R.M., Wellenstein K., Keefe W., Schickler M. (2011). PD-1 Blockade by CT-011, Anti-PD-1 Antibody, Enhances Ex Vivo T-cell Responses to Autologous Dendritic Cell/Myeloma Fusion Vaccine. J. Immunother..

[5] Zelle-Rieser C., Thangavadivel S., Biedermann R., Brunner A., Stoitzner P., Willenbacher E., Greil R., Jöhrer K. (2016). T cells in multiple myeloma display features of exhaustion and senescence at the tumor site. J. Hematol. Oncol..

[6] Hallett W.H., Jing W., Drobyski W.R., Johnson B.D. (2011). Immunosuppressive Effects of Multiple Myeloma Are Overcome by PD-L1 Blockade. Biol. Blood Marrow Transplant..

[7] Xing J., Lu G., Liu G.Q., Xu M., Zhao X., Han F., Wang L., Ding H.F. (2014). Imbalance of treg/th17 in bone marrow of patients with multiple myeloma. Zhongguo Shi Yan Xue Ye Xue Za Zhi.

[8] Atanackovic D., Luetkens T. (2018). Biomarkers for checkpoint inhibition in hematologic malignancies. Semin. Cancer Biol..

[9] Kearl T.J., Jing W., Gershan J.A., Johnson B.D. (2013). Programmed Death Receptor-1/Programmed Death Receptor Ligand-1 Blockade after Transient Lymphodepletion To Treat Myeloma. J. Immunol..

[10] Ansell S.M., Lesokhin A.M., Borrello I., Halwani A., Scott E.C., Gutierrez M., Schuster S.J., Millenson M.M., Cattry D., Freeman G.J. (2015). PD-1 Blockade with Nivolumab in Relapsed or Refractory Hodgkin’s Lymphoma. New Engl. J. Med..

[11] Thanendrarajan S., Puryear J., Schinke C.D., van Rhee F., Zangari M., Mathur P., Mohan M., Susanibar S., Kamimoto J.J., Hoque S. (2017). Nivolumab for treatment of advanced, refractory, high-risk multiple myeloma. Blood.

[12] Suen H., Brown R.F., Yang S., Ho P.J., Gibson J., Joshua D. (2015). The failure of immune checkpoint blockade in multiple myeloma with PD-1 inhibitors in a phase 1 study. Leukemia.

[13] Suen H., Brown R., Yang S., Weatherburn C., Ho P.J., Woodland N., Nassif N., Barbaro P., Bryant C., Hart D. (2016). Multiple myeloma causes clonal T-cell immunosenescence: Identification of potential novel targets for promoting tumour immunity and implications for checkpoint blockade. Leukemia.

[14] Zhao Y., Shao Q., Peng G. (2019). Exhaustion and senescence: Two crucial dysfunctional states of T cells in the tumor microenvironment. Cell. Mol. Immunol..

[15] Pawelec G. (2019). Is There a Positive Side to T Cell Exhaustion?. Front. Immunol..

[16] Kosmaczewska A., Masternak A., Kosciow K., Ciszak L., Usnarska-Zubkiewicz L., Szteblich A., Potoczek S., Frydecka I. (2019). PD-1 overexpression determines the disproportion of circulating Th1/Th17/Treg cells and clinical outcome of multiple myeloma. Res. Sq..

[17] Pardoll D.M. (2012). The blockade of immune checkpoints in cancer immunotherapy. Nat. Rev. Cancer.

[18] Kosmaczewska A., Frydecka I., Boćko D., Ciszak L., Teodorowska R. (2002). Correlation of blood lymphocyte CTLA-4 (CD152) induction in Hodgkin’s disease with proliferative activity, interleukin 2 and interferon-gamma production. Br. J. Haematol..

[19] Frydecka I., Kosmaczewska A., Bocko D., Ciszak L., Wolowiec D., Kuliczkowski K., Kochanowska I. (2004). Alterations of the expression of T-cell related costimulatory CD28 and down regulatory CD152 (CTLA-4) molecules in patients with B-cell chronic lymphocytic leukemia. Br. J. Cancer.

[20] Kosmaczewska A., Ciszak L., Suwalska K., Wolowiec D., Frydecka I. (2004). CTLA-4 overexpression in CD19^+^/CD5^+^ cells correlates with the level of cell cycle regulators and disease progression in B-CLL patients. Leukemia.

[21] Ciszak L., Frydecka I., Wolowiec D., Szteblich A., Kosmaczewska A. (2016). CTLA-4 affects expression of key cell cycle regulators of G0/G1 phase in neoplastic lymphocytes from patients with chronic lymphocytic leukaemia. Clin. Exp. Med..

[22] Karabon L., Partyka A., Ciszak L., Pawlak-Adamska E., Tomkiewicz A., Bojarska-Junak A., Roliński J., Wołowiec D., Wrobel T., Frydecka I. (2020). Abnormal Expression of BTLA and CTLA-4 Immune Checkpoint Molecules in Chronic Lymphocytic Leukemia Patients. J. Immunol. Res..

[23] Ciszak L., Frydecka I., Wolowiec D., Szteblich A., Kosmaczewska A. (2016). Patients with chronic lymphocytic leukaemia (CLL) differ in the pattern of CTLA-4 expression on CLL cells: The possible implications for immunotherapy with CTLA-4 blocking antibody. Tumour Biol..

[24] Thommen D.S., Schreiner J., Müller P., Herzig P., Roller A., Belousov A., Umana P., Pisa P., Klein C.G., Bacac M. (2015). Progression of Lung Cancer Is Associated with Increased Dysfunction of T Cells Defined by Coexpression of Multiple Inhibitory Receptors. Cancer Immunol. Res..

[25] Tan J., Chen S., Huang J., Chen Y., Yang L., Wang C., Zhong J., Lu Y., Wang L., Zhu K. (2018). Increased exhausted CD8^+^T cells with programmed death-1, T-cell immunoglobulin and mucin-domain-containing-3 phenotype in patients with multiple myeloma. Asia-Pacific J. Clin. Oncol..

[26] Arai Y., Saito H., Ikeguchi M. (2012). Upregulation of TIM-3 and PD-1 on CD4^+^ and CD8^+^ T Cells Associated with Dysfunction of Cell-Mediated Immunity after Colorectal Cancer Operation. Yonago Acta Med..

[27] Coppé J.-P., Desprez P.-Y., Krtolica A., Campisi J. (2010). The Senescence-Associated Secretory Phenotype: The Dark Side of Tumor Suppression. Annu. Rev. Pathol. Mech. Dis..

[28] Pauken K.E., Wherry E.J. (2015). Overcoming T cell exhaustion in infection and cancer. Trends Immunol..

[29] Liu X., Mo W., Ye J., Li L., Zhang Y., Hsueh E.C., Hoft D.F., Peng G. (2018). Regulatory T cells trigger effector T cell DNA damage and senescence caused by metabolic competition. Nat. Commun..

[30] Ye J., Huang X., Hsueh E.C., Zhang Q., Ma C., Zhang Y., Varvares M.A., Hoft D.F., Peng G. (2012). Human regulatory T cells induce T-lymphocyte senescence. Blood.

[31] Rajkumar S.V., Dimopoulos M.A., Palumbo A., Blade J., Merlini G., Mateos M.-V., Kumar S., Hillengass J., Kastritis E., Richardson P. (2014). International Myeloma Working Group updated criteria for the diagnosis of multiple myeloma. Lancet Oncol..

[32] Greipp P.R., Miguel J.S., Durie B.G., Crowley J.J., Barlogie B., Bladé J., Boccadoro M., Child J.A., Avet-Loiseau H., Kyle R.A. (2005). International Staging System for Multiple Myeloma. J. Clin. Oncol..

[33] Liu W., Putnam A.L., Xu-Yu Z., Szot G.L., Lee M.R., Zhu S., Gottlieb P.A., Kapranov P., Gingeras T.R., Fazekas de St Groth B. (2006). CD127 expression inversely correlates with FoxP3 and suppressive function of human CD^4+^ T reg cells. J. Exp. Med..

[34] Petersen C., Christensen E., Andresen B., Møller B. (1992). Internalization, lysosomal degradation and new synthesis of surface membrane CD4 in phorbol ester-activated T-lymphocytes and U-937 cells. Exp. Cell Res..

[35] Batorov E.V., Aristova T.A., Sergeevicheva V.V., Sizikova S.A., Ushakova G.Y., Pronkina N.V., Shishkova I.V., Shevela E.Y., Ostanin A.A., Chernykh E.R. (2020). Quantitative and functional characteristics of circulating and bone marrow PD-1- and TIM-3-positive T cells in treated multiple myeloma patients. Sci. Rep..

[36] Montes C.L., Chapoval A.I., Nelson J., Orhue V., Zhang X., Schulze D.H., Strome S.E., Gastman B.R. (2008). Tumor-Induced Senescent T Cells with Suppressor Function: A Potential Form of Tumor Immune Evasion. Cancer Res..

[37] Ye J., Peng G. (2015). Controlling T cell senescence in the tumor microenvironment for tumor immunotherapy. OncoImmunology.

[38] Ye J., Ma C., Hsueh E.C., Dou J., Mo W., Liu S., Han B., Huang Y., Zhang Y., Varvares M.A. (2014). TLR 8 signaling enhances tumor immunity by preventing tumor-induced T-cell senescence. EMBO Mol. Med..

[39] Lee M.Y., Park C.-J., Cho Y.-U., Jang S. (2019). PS1426 IMMUNE CHECKPOINT (PD-1, PD-L1, PD-L2, AND CTLA-4) EXPRESSION IN PLASMA CELL MYELOMA. HemaSphere.

[40] Simonetta F., Pradier A., Bosshard C., Masouridi-Levrat S., Dantin C., Koutsi A., Tirefort Y., Roosnek E., Chalandon Y. (2019). Dynamics of Expression of Programmed Cell Death Protein-1 (PD-1) on T Cells After Allogeneic Hematopoietic Stem Cell Transplantation. Front. Immunol..

[41] Lesokhin A.M., Ansell S.M., Armand P., Scott E.C., Halwani A., Gutierrez M., Millenson M.M., Cohen A.D., Schuster S.J., Lebovic D. (2014). Preliminary Results of a Phase I Study of Nivolumab (BMS-936558) in Patients with Relapsed or Refractory Lymphoid Malignancies. Blood.

[42] Lesokhin A.M., Ansell S.M., Armand P., Scott E.C., Halwani A., Gutierrez M., Millenson M.M., Cohen A.D., Schuster S.J., Lebovic D. (2016). Nivolumab in Patients With Relapsed or Refractory Hematologic Malignancy: Preliminary Results of a Phase Ib Study. J. Clin. Oncol..

[43] Webb E.S., Liu P., Baleeiro R., Lemoine N.R., Yuan M., Wang Y.-H. (2017). Immune checkpoint inhibitors in cancer therapy. J. Biomed. Res..

[44] Kao C., Oestreich K.J., Paley M.A., Crawford A., Angelosanto J.M., Ali M.A., Intlekofer A.M., Boss J.M., Reiner S.L., Weinmann A.S. (2011). Transcription factor T-bet represses expression of the inhibitory receptor PD-1 and sustains virus-specific CD8^+^ T cell responses during chronic infection. Nat. Immunol..

[45] Lu P., Youngblood B., Austin J.W., Mohammed A.U.R., Butler R., Ahmed R., Boss J.M. (2014). Blimp-1 represses CD8 T cell expression of PD-1 using a feed-forward transcriptional circuit during acute viral infection. J. Exp. Med..

[46] Kasamatsu T., Awata M., Ishihara R., Murakami Y., Gotoh N., Matsumoto M., Sawamura M., Yokohama A., Handa H., Tsukamoto N. (2019). PDCD1 and PDCD1LG1 polymorphisms affect the susceptibility to multiple myeloma. Clin. Exp. Med..

[47] Karabon L., Pawlak-Adamska E., Tomkiewicz A., Jedynak A., Kielbinski M., Woszczyk D., Potoczek S., Jonkisz A., Kuliczkowski K., Frydecka I. (2011). Variations in Suppressor Molecule CTLA-4 Gene Are Related to Susceptibility to Multiple Myeloma in a Polish Population. Pathol. Oncol. Res..

[48] Zheng C., Huang D., Liu L., Björkholm M., Holm G., Yi Q., Sundblad A. (2001). Cytotoxic T-lymphocyte antigen-4 microsatellite polymorphism is associated with multiple myeloma. Br. J. Haematol..

[49] Alrasheed N., Lee L., Ghorani E., Henry J.Y., Conde L., Chin M., Galas-Filipowicz D., Furness A.J., Chavda S.J., Richards H. (2020). Marrow-Infiltrating Regulatory T Cells Correlate with the Presence of Dysfunctional CD4^+^PD-1+ Cells and Inferior Survival in Patients with Newly Diagnosed Multiple Myeloma. Clin. Cancer Res..

[50] Usmani S.Z., Schjesvold F., Rocafiguera A.O., Karlin L., Rifkin R.M., Yimer H.A., Leblanc R., Takezako N., McCroskey R.D., Suzuki K. (2018). A phase 3 randomized study of pembrolizumab (pembro) plus lenalidomide (len) and low-dose dexamethasone (Rd) versus Rd for newly diagnosed and treatment-naive multiple myeloma (MM): KEYNOTE-185. J. Clin. Oncol..

[51] Mateos M.-V., Blacklock H., Schjesvold F., Rocafiguera A.O., Simpson D., George A., Goldschmidt H., LaRocca A., Sherbenou D.W., Avivi I. (2018). A phase 3 randomized study of pembrolizumab (Pembro) plus pomalidomide (Pom) and dexamethasone (Dex) for relapsed/refractory multiple myeloma (RRMM): KEYNOTE-183. J. Clin. Oncol..

[52] Tamura H., Ishibashi M., Sunakawa-Kii M., Inokuchi K. (2020). PD-L1–PD-1 Pathway in the Pathophysiology of Multiple Myeloma. Cancers.

[53] Ishibashi M., Tamura H., Sunakawa M., Kondo-Onodera A., Okuyama N., Hamada Y., Moriya K., Choi I., Tamada K., Inokuchi K. (2016). Myeloma Drug Resistance Induced by Binding of Myeloma B7-H1 (PD-L1) to PD-1. Cancer Immunol. Res..

[54] Amarnath S., Mangus C.W., Wang J.C.M., Wei F., He A., Kapoor V., Foley J.E., Massey P.R., Felizardo T.C., Riley J. (2011). The PDL1-PD1 Axis Converts Human TH1 Cells into Regulatory T Cells. Sci. Transl. Med..

[55] Prabhala R.H., Pelluru D., Fulciniti M., Prabhala H.K., Nanjappa P., Song W., Pai C., Amin S., Tai Y.-T., Richardson P.G. (2010). Elevated IL-17 produced by Th17 cells promotes myeloma cell growth and inhibits immune function in multiple myeloma. Blood.

[56] Braga W.M., da Silva B.R., de Carvalho A.C., Maekawa Y.H., Bortoluzzo A.B., Rizzatti E.G., Atanackovic D., Colleoni G.W. (2014). FOXP3 and CTLA4 overexpression in multiple myeloma bone marrow as a sign of accumulation of CD4(^+^) T regulatory cells. Cancer Immunol Immunother..

[57] Favaloro J., Brown R., Aklilu E., Yang S., Suen H., Hart D., Fromm P., Gibson J., Khoo L., Ho P.J. (2013). Myeloma skews regulatory T and pro-inflammatory T helper 17 cell balance in favor of a suppressive state. Leuk. Lymphoma.

