# Supplementary material for: Deregulated Expression of Immune Checkpoints on Circulating CD4 T Cells May Complicate Clinical Outcome and Response to Treatment with Checkpoint Inhibitors in Multiple Myeloma Patients
Source: Int J Mol Sci. 2021 Aug 27;22(17):9298. doi: 10.3390/ijms22179298 (PMC8431347; doi:10.3390/ijms22179298)
Supplement: Supplementary file 1 [file ijms-22-09298-s001.zip › ijms-1331761-supplementary.pdf]

Table S1. Patient demographics and characteristics.

Characteristic	Number/frequency
Number of patients, n (%)	40
Gender (female)	21 (52.5 %)
Age of sampling (median, range)	69 (59–76)
ISS	
I	6 (15.0 %)
II	16 (40.0 %)
III	18 (45.0 %)
Myeloma isotype	
IgG	27 (67.5 %)
IgA	6 (15.0 %)
Light chain only	7 (17.5 %)
Type of Ig light chain (serum)	
Kappa	23 (57.5 %)
Lambda	16 (40.0 %)
None	1 (2.5 %)
Osteolytic bone lesion/s, n (%)	28 (70.0 %)
Laboratory values	
β2-microglobulin ≥ 3.5 mg/l	27 (67.5 %)
Creatinine ≥ 2.0 mg/dl	13 (32.5 %)
LDH > 190 U/L	8 (20.0 %)
Serum calcium ≥ 10 mg/dl	20 (50.0 %)
Hemoglobin ≤ 12 g/dl	31 (80.0 %)
Platelets < 100,000/mm^3	3 (7.5 %)
Prior treatment	
1-3 therapy lines	8 (20.0 %)
≥ 4 therapy lines	6 (15.0 %)
BTZ based therapy	12 (30.0 %)
IMiD therapy	11 (27.5 %)
No therapy	26 (65.0 %)

Abbreviations: BTZ, bortezomib; LDH, lactate dehydrogenases; ISS, International Staging System; IMiD, immunomodulatory drugs; UNV, upper normal values

Table S2. Immune checkpoint and CD69 expression (%) in PB CD4 T cell subsets in MM stages and controls (HC).

T-cell subset (%)	Stage I/II of MM	Stage III of MM	HC	*P* - value
	(n = 22)	(n = 18)	(n =20)	
$CD3^+CD4^+PD\text{-}1^+$	16.8 (11.68-19.86)	14.7 (12.18-18.87)	12.54 (8.37-15.29)	a) ns b) 0.012 c) 0.08*
$CD4^+CD127^+PD\text{-}1^+$	23.4 (16.57-30.05)	22.2 (13.97-31.42)	16.8 (10.52-21.75)	a) ns b) 0.023 c) 0.027
$CD4^+CD127^-PD\text{-}1^+$	9.70 (6.44-13.98)	11.49 (7.97-12.80)	6.14 (3.90-7.62)	a) ns b) 0.023 c) 0.027
$CD3^+CD4^+BTLA^+$	27.38 (2.46-4.76)	26.45 (2.38-5.58)	23.85 (17.04-40.16)	a) ns b) ns

				c) ns
$CD4^+CD127^+BTLA^+$	23.30 (13.29-49.21)	24.78 (15.60-51.60)	32.50 (20.75-54.43)	a) ns b) ns c) ns
$CD4^+CD127^-BTLA^+$	5.10 (2.06-7.60)	4.05 (3.72-7.35)	2.87 (1.85-3.40)	a) ns b) ns c) ns
$CD3^+CD4^+CTLA\text{-}4^+$	1.61 (1.00-2.51)	1.17 (0.55-3.04)	1.27 (0.78-2.00)	a) ns b) ns c) ns
$CD4^+CD127^+CTLA4^+$	3.28 (2.21-6.00)	3.84 (1.47-6.07)	3.55 (1.64-4.22)	a) ns b) ns c) ns
$CD4^+CD127^-CTLA4^+$	1.57 (0.75-2.20)	1.99 (0.88-2.49)	1.14 (0.56-1.37)	a) ns b) ns c) ns
$CD3^+CD4^+CD69^+$	0.56 (0.22-0.83)	0.94 (0.35-1.21)	0.42 (0.23-0.44)	a) 0.08* b) ns c) 0.006

a) Stage I/II vs. stage III
b) Stage I/II vs. HC
c) Stage III vs. HC
*trend; ns – not statistically significant

Differences in the proportions of PD-1, BTLA, and CTLA-4 expressing CD4+ T cells between examined groups were evaluated using nonparametric tests (Kruskal-Wallis and Mann-Whitney U-test).

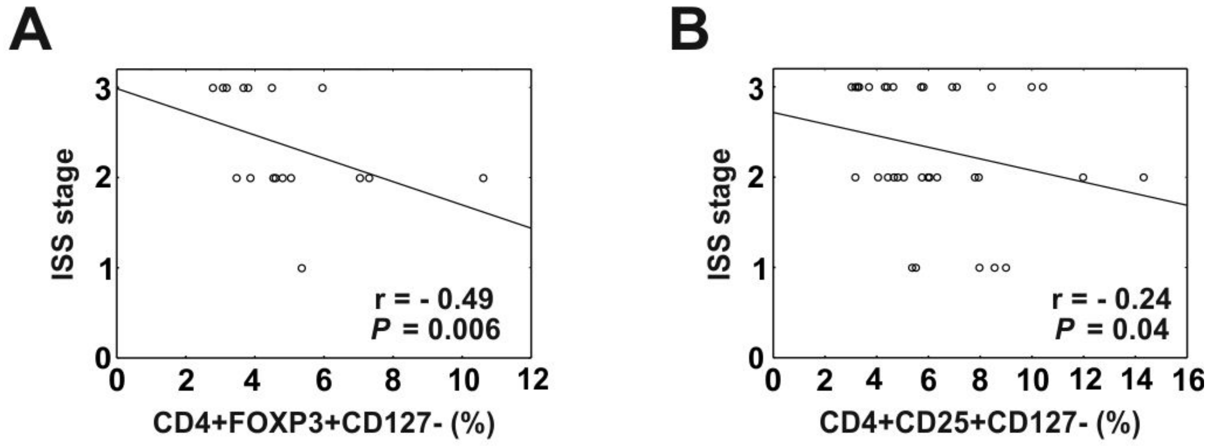

FIGURE S1: Correlation of CD127- Treg subsets with clinical stages of myeloma. We found negative associations between the abundance of PB Treg cells and MM stage.

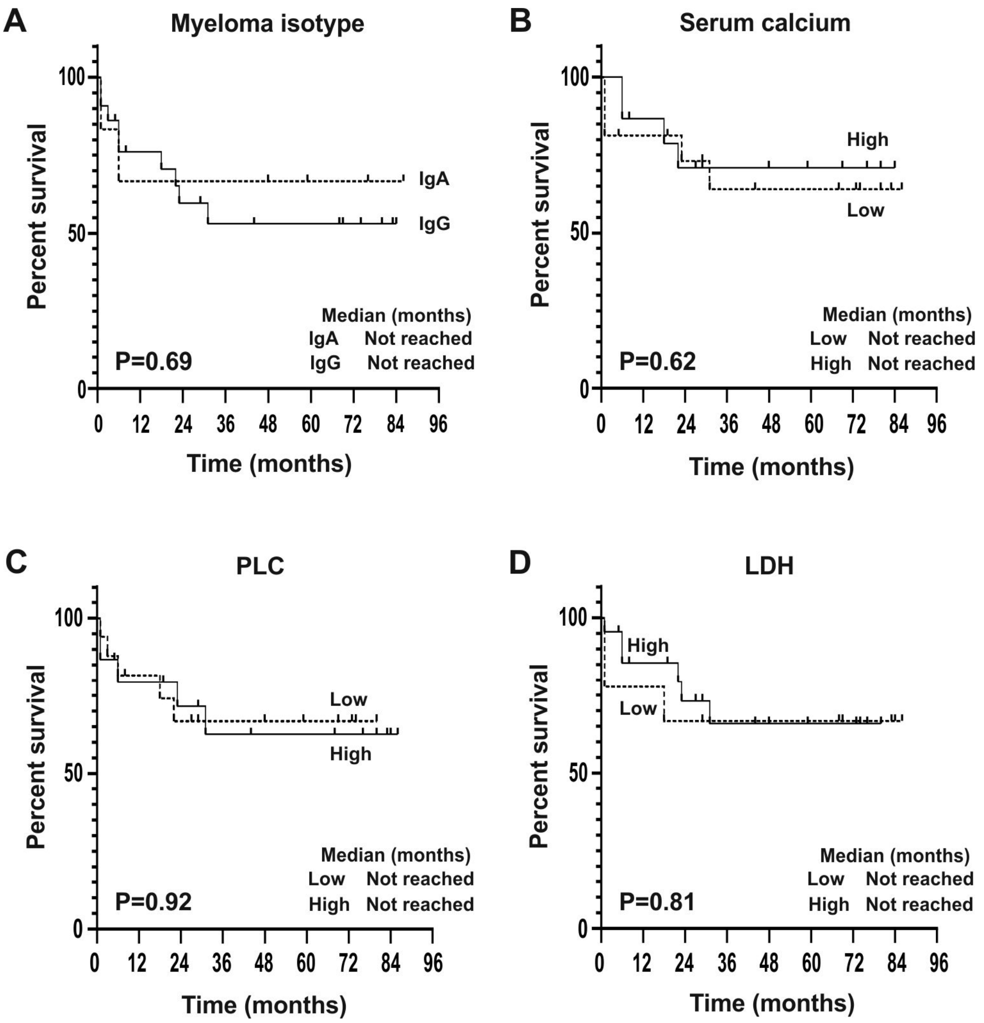

FIGURE S2: Effect of clinical features on patient survival. The cohort of studied patients was divided into high and low expressors of clinical indices according to the median split. We found no significant influence of (A) myeloma isotype, (B) serum calcium level, (C) circulating plasmocytes (PLC), or (D) LDH activity on patient survival ($p > 0.05$). We used the log-rank test in statistical comparison.
